# Who gets a lung transplant? Assessing the psychosocial decision-making process for transplant listing

**DOI:** 10.21542/gcsp.2016.26

**Published:** 2016-09-30

**Authors:** Amber N. Lewandowski, Jared Lyon Skillings

**Affiliations:** Richard DeVos Heart and Lung Transplant Program, Spectrum Health, Grand Rapids, MI, USA

**Keywords:** Organ transplant, psychosocial assessment, organ allocation, bias, transplant selection criteria, transplant listing

## Abstract

In the United States, there is a significant shortage of available donor organs. This requires transplant professionals to hold simultaneous, yet divergent roles as (1) advocates for patients who are in need of a lifesaving transplant, and (2) responsible stewards in the allocation of scarce donor organs. In order to balance these roles, most transplant teams utilize a committee based decision-making process to select suitable candidates for the transplant waiting list. These committees use medical and psychosocial criteria to guide their decision to list a patient. Transplant regulatory bodies have established medical standards for identifying appropriate medical candidates for transplantation. However, transplant regulatory bodies have not developed policies to standardize psychosocial criteria for listing patients. This affords transplant centers the autonomy to develop their own psychosocial criteria for determining which patients will be placed on the transplant waiting list. This lack of a standardized policy has resulted in inconsistent psychosocial practices amongst transplant centers nationwide. Since there has been no formal review of the inconsistency in psychosocial policy and practice, this paper seeks to explore the non-standardized psychosocial approach to organ transplant listing. The authors review factors that are relevant to the standardization of the psychosocial decision-making process, including shared decision-making, clinician judgment, bias in decision-making and moral distress in transplant staff. We conclude with a discussion about the impact of these issues on psychosocial practices in solid organ transplantation.

## Introduction

When a patient is experiencing end-stage lung disease one treatment option that may be available is a lung transplant. While transplantation may be a treatment option, a patient must undergo a thorough evaluation to determine if they are a suitable candidate for a transplant. If a patient is an appropriate candidate they are then placed on a waiting list. Both medical and psychosocial factors are considered when determining a patient’s candidacy for transplantation and placement on the waiting list. This article reviews one aspect of the decision-making process for listing patients: the psychosocial aspect. To date, transplant regulatory bodies in the United States have not established universal psychosocial assessment or listing criteria. This has afforded transplant centers the autonomy to develop their own psychosocial criteria that patients must meet in order to be placed on the transplant waiting list. This has resulted in inconsistency of psychosocial practices amongst transplant centers nationwide. Therefore, the current approach to psychosocial assessment and listing criteria is non-standardized.

To the best of the authors’ knowledge, there is no article that reviews the psychosocial decision-making process for lung transplant listing. We provide an exploratory review of this issue as a way to initiate professional dialogue within the transplant community about the role of psychosocial assessments and listing criteria. In order to understand the psychosocial aspects of the decision-making process, we start by describing the organ allocation system, process of patient listing, transplant regulations, and psychosocial complexities of the decision-making process. We then review factors that are relevant to the standardization of the psychosocial decision-making process, including shared decision-making, clinician judgment, bias in decision-making and moral distress in transplant staff, and conclude with a discussion about the impact of these issues on psychosocial practices in solid organ transplantation.

Since there is very limited data regarding the psychosocial aspects of lung transplantation that impact listing decisions, we utilize research from a variety of solid organ transplant literature. The authors believe that psychosocial expectations should be largely uniform for transplant patients, regardless of the transplanted organ. Thus, the use of data from a variety of solid organ transplant literature is consistent with the goals of this paper, and we envision this to be useful not only for lung patients but also for other solid organ transplant candidates. However, with the introduction of intermediary therapies, such as dialysis for patients with end-stage renal disease or ventricular assist devices for patients with heart failure that can extend a patient’s life while they wait for a transplant, there may be some unique differences in psychosocial considerations depending on the type of organ failure.

## Organ allocation system and the process of patient listing

In the United States, there are currently over 121,000 individuals waiting for an organ transplant^1^. However, only 24,982 deceased donations occurred in 2015^2^. Given these statistics, it is clear that there is a greater need for solid organ transplants than there are available donor organs. One way in which the United States has worked to address this shortage is through a nationwide system of organ allocation, which matches patients who are the sickest and thus in greatest need of a transplant with available donor organs. This system was established by the National Organ Transplant Act (NOTA) of 1984^3^. The act called for an Organ Procurement and Transplantation Network (OPTN) that would provide national oversight of the organ donation process. In 1986, the Department of Health and Human Services awarded the OPTN contract to the United Network for Organ Sharing (UNOS). The OPTN is responsible for the nationwide system that matches available donor organs to transplant candidates. It also creates and maintains policies for transplant centers, in order to ensure the equitable distribution of donor organs and increase community access to transplantation^4^.

The OPTN and the Centers for Medicare and Medicaid Services (CMS) are the primary governing entities regarding the practices of transplant centers in the United States. Transplant programs are required to follow a number of regulatory guidelines developed by these establishments. Additionally, the International Society for Heart and Lung Transplantation (ISHLT) is the leading professional association for heart and lung transplant programs, which establishes best practices in the field of transplantation. A review of the respective policies and recommendations of each organization provides broad guidance on the psychosocial aspects of patient care and patient selection for transplant listing.

The OPTN bylaws require transplant programs to have designated members of the transplant team who can provide psychiatric and social services^5^. It is the responsibility of these professionals to evaluate and manage patients’ psychosocial needs and aid in the continuity of care. Standard services include psychosocial evaluation; substance abuse evaluation, treatment, referral, and monitoring; individual counseling; crisis intervention; patient advocacy; patient and family education; and community referrals. For the selection of transplant candidates, the OPTN allows transplant programs to create their own policies and procedures providing that they are consistent with the OPTN bylaws.

CMS is another organization that directs the practices of transplant centers. CMS establishes conditions of participation under which transplant programs will be reimbursed for services provided to patients who have Medicare and/or Medicaid insurance. In the United States, the majority of private insurance companies institute similar practices as CMS for reimbursement of their services rendered. CMS regulations complement OPTN bylaws by requiring a standard psychosocial evaluation for all potential transplant candidates, as long as the patient’s medical condition is stable enough to participate in an evaluation^6^. Furthermore, a qualified social worker is required to provide social services based on patient needs. While CMS regulations require a psychosocial evaluation that focuses on patient suitability for transplantation, they do not identify a standardized approach to the evaluation, nor do they specify what role psychosocial factors should play in the decision-making process for listing transplant candidates.

Finally, while not considered a regulatory body, the ISHLT Pulmonary Transplantation Council has established recommendations for the selection of lung transplant candidates. These recommendations reflect a committee consensus of expert opinion in the field of transplantation for medical and psychosocial aspects of patient care^7^. In comparison to OPTN bylaws and CMS regulations, more guidance is offered on the selection of lung transplant candidates based on psychosocial characteristics. The committee identifies absolute psychosocial contraindications, such as chronic non-adherence, severe psychiatric conditions, unreliable social support, and active substance abuse (e.g. alcohol, tobacco, and other illicit substances) that would lead to a poor outcome post-transplantation. However, the committee does not consider these recommendations as definitive. In fact, they caution against the use of these recommendations as the standard of care in lung transplantation.

## Transplant selection methods

Patients are initially identified as potential transplant candidates when they are referred by their primary care physician or another specialist to a transplant center for assessment of their end-stage organ disease. A patient then undergoes a comprehensive medical and psychosocial evaluation by a transplant team—a multidisciplinary group of specially trained professionals. In the case of lung transplantation, the team would likely include a pulmonologist, cardiothoracic surgeon, dietician, financial coordinator, nurse practitioner or physician’s assistant, nurse coordinator, pharmacist, psychologist/psychiatrist, and social worker.

Once a patient has completed their evaluation, the selection process for listing on the transplant waiting list begins. Typically, a nurse coordinator organizes test results and team members’ recommendations into a summary of the patient’s potential risks and protective factors. This information is then presented to a committee of health care professionals who select patients for placement on the transplant waiting list. For the purpose of this paper, these committees will be referred to as selection committees. Selection committees are the preferred method by which transplant programs allocate scarce donor organs and achieve optimal patient outcomes. They are primarily comprised of members of the evaluating transplant team. This necessarily places transplant professionals in a difficult position where they must advocate for their patients who are in need of a lifesaving transplant, while also serving as responsible stewards in the allocation of a scarce resource.

The primary function of transplant selection committees is to determine if a patient is (1) suitable for listing on the transplant waiting list, (2) deferred from listing until medical and/or psychosocial concerns are resolved, or (3) not a candidate due to unresolvable medical and/or psychosocial concerns. In the decision to select a patient for transplantation, most programs use psychosocial information to determine a patient’s psychosocial stability and ability to adhere to a complex, lifelong regimen of care; this is an evaluative, gate-keeping function. Simultaneously, many programs use this information to identify psychosocial concerns and provide targeted interventions to improve a patient’s candidacy and adherence behaviors^8–10^; this is a rehabilitative or advocacy function. The decision-making process for listing patients differs by program. Some transplant programs prefer a voting method or group consensus, while other programs rely upon a physician-led decision.

## Psychosocial complexities of transplant listing

**Figure 1.**
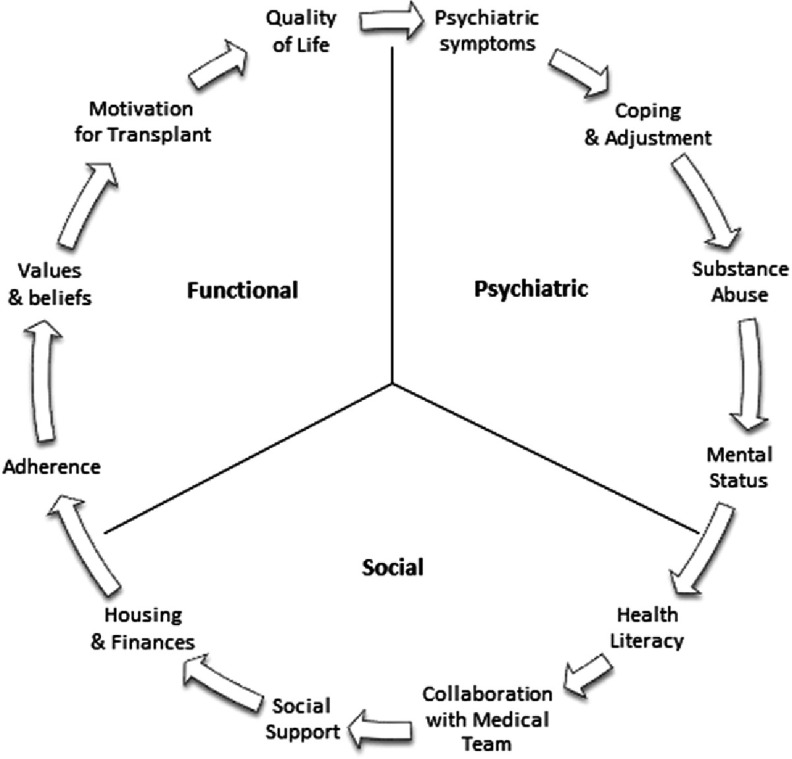
Key components of the transplant psychosocial assessment.

To act in accordance with existing regulations, transplant programs develop their own policies for the evaluation and selection of suitable candidates for listing. As highlighted previously, the decision to place a patient on the transplant waiting list is not exclusively based on medical criteria. Psychosocial factors are typically considered in the process of selecting patients, which adds complexity to the decision-making process. In a 2015 paper, the authors described the key interconnected components of a psychosocial transplant evaluation, which fall into three domains: psychiatric, social, and functional^11^. The psychiatric portion of the evaluation focuses on psychiatric history and current stability; coping mechanisms; general adjustment; recent and past substance abuse, including nicotine and alcohol use; and mental status. The social portion of the evaluation examines health literacy; ability to collaborate with the transplant team and family/caregivers in their care; quality of social support; and stability of housing and finances. Finally, the functional aspect assesses recent and past adherence behaviors; individual values and beliefs; understanding of and motivation for transplantation; and quality of life (see Figure 1).

 To illustrate the complexity of the selection process regarding psychosocial factors, we include a brief case study of Angela. This description is followed by a review of the psychosocial factors involved in this case, along with applicable research that would likely be considered by a selection committee.

Angela was a 40-year-old woman who was admitted to the hospital with acute symptoms of idiopathic pulmonary fibrosis, requiring an emergent transplant evaluation. On review of her electronic health record, it was found that she generally took her medications, occasionally attended medical appointments, and was a heavy cannabis user. Upon admission to the hospital, her serum toxicology screen was negative for illicit drugs except for cannabis. During the transplant evaluation, Angela was engaging and interpersonally stable. Her family was at her bedside throughout the evaluation process. They appeared highly supportive and invested in her care. Her mother reassured the transplant team that her family would make sure that Angela would take all of her medications and attend medical appointments in the future. Angela expressed a strong desire to live, and she promised to follow all of the program rules if they could “save my life with a transplant.”

This case illustrates three prominent psychosocial factors that a selection committee would likely consider in their discussion: (1) adherence, (2) substance use, and (3) social support. Below we provide a brief review of each factor including research from the organ transplant literature.

First regarding adherence, it is important to note that there are a number of requirements for a patient after receiving an organ transplant. Patients must take lifelong immunosuppressant medication; attend frequent outpatient medical appointments; participate in routine blood draws; and maintain overall health through dietary choices, exercise, and abstinence from substance use/abuse. Nonadherence to this transplant regimen can lead to serious consequences. For example, nonadherence to immunosuppressant medication has been related to higher rates of acute rejection, graft dysfunction, and mortality^12^. There is limited data on the rates of adherence with immunosuppressant medication in lung transplant recipients. In one study of lung recipients, however, approximately 13% showed persistent nonadherence over a two-year period^13^. This is consistent with the average of 14.3 cases per 100 solid organ transplant recipients per year that are nonadherent to their immunosuppressant medications^14^.

Angela’s second risk factor is substance use, since she is a heavy cannabis user. There has been significant debate within the transplant community regarding patients’ use of cannabis. A survey of transplant programs showed that 37.5% required 6 months of abstinence prior to listing, 18.5% required 3 months of abstinence prior to listing, 25% did not require a period of abstinence, and 12.5% did not have a clear policy on marijuana use^15^. These differences in practice may be partly due to the fact that there have been few studies on cannabis use in transplantation. The two potential risks for cannabis use that are highlighted most frequently in the literature are fungal infections in immunosuppressed patients and an increased risk of other addictions, mental illness, or medical illness like chronic bronchitis^16,17^. Due to the scarcity of research at this point in time, such risks are considered to be potential rather than definitive. Most studies have found no evidence that cannabis use leads to poorer transplant outcomes^18,19^.

Third, Angela’s support system appears to be a notable protective factor. Research has demonstrated a strong relationship between good social support, medical adherence rates, and overall health outcomes^20–22^. Similarly, poor social support has been identified as a substantial risk factor for survival post-transplantation and predictor of graft failure^23^. It is important to note that the concept of social support includes both emotional and/or instrumental support factors, both of which can serve a protective function for transplant patients^24^.

If a transplant selection committee were to review Angela’s case, their task would be to determine if her two psychosocial risk factors, independently or combined, are significant enough to be considered prohibitive risks for transplantation. The committee’s decision-making process would likely also review Angela’s protective factors to determine the extent to which they may minimize her risk or enhance her health. This is a complex decisional matrix. Angela has been occasionally nonadherent and is a heavy cannabis user, yet she has a strong support network who promises to aid in her post-surgical adherence. Each one of these factors could influence Angela’s adjustment and ability to care for a transplant. For instance, Angela’s family may be able to provide necessary practical and emotional support as she works through her sobriety and surgical recovery. At the same time, the question arises as to whether Angela’s social support would be enough to help her maintain lifelong sobriety and behavioral changes that would promote her success with a lung transplant.

National regulatory bodies, such as the OPTN and CMS, do not provide detailed guidance about whether a particular patient, like Angela, should be considered a prohibitory transplant risk. Unfortunately, the ISHLT that develops best practice guidelines for transplant professionals also does not offer detailed approaches to such questions, nor does it define psychosocial terminology like ‘non-adherence’ or ‘substance abuse.’ Moreover, the psychosocial transplant literature is limited regarding outcomes of single psychosocial variables (like cannabis use), and scarce for complex, multi-factorial psychosocial circumstances. Consequently, this has led to a non-standardized, or in other words variable, decision-making approach to psychosocial criteria for transplant programs in the United States.

## Non-standardized psychosocial listing criteria

While the majority of transplant centers use psychosocial factors in the decision-making process for transplant listing, specific data on psychosocial listing factors for lung transplant programs is absent in the current literature. However in a survey of heart transplant programs, 99% reported that psychosocial criteria was considered in the process of selecting a patient for the transplant waiting list, and the proportion of patients declined for transplant listing based on psychosocial reasons ranged from 0% to 37.5%^25^. In fact, 92% of the programs surveyed in the United States reported that they would not select a patient for listing unless a social worker, psychologist, or psychiatrist considered the patient to be a suitable candidate. Thus, the standardization of psychosocial evaluation criteria would affect a substantial number of transplant programs and patients. We therefore include a review of the following variables that are relevant to the standardization of the psychosocial decision-making process: (1) shared decision-making, (2) clinician judgment, (3) bias in decision-making and (4) moral distress in transplant staff.

First, there are three forms of decision-making models in health care: paternalistic, shared, and informed^26^. In a paternalistic decision-making model, a clinician ultimately makes the decisions in a patient’s care; whereas, in a shared decision-making model, the clinician and patient make decisions together. With an informed decision-making model, patients make the final decision about their health care. Both the shared and informed decision-making models are thus fundamentally patient-centered. Arguably, a strict assessment or set of psychosocial listing criteria favors a paternalistic decision-making model. However, patients have reported a variety of preferences with regard to decision-making approaches. In one study, for example, 52% of respondents reported that they would prefer their physician make final decisions in their health care^27^. Thus, it cannot be assumed that patients universally prefer one decision-making method or that certain decision-making methods lead to better medical outcomes.

Second, the use of clinical judgment versus statistical prediction for the selection of transplant candidates requires examination. In one meta-analysis, it was found that the use of statistical prediction improved clinician judgments by approximately 10%^28^. To date there have been no known similar studies in organ transplantation. However, there are risk assessment tools for transplantation that attempt to estimate a patient’s level of psychosocial risk, which in turn could affect their recovery or success with a transplant. These tools include the Psychosocial Assessment of Candidates for Transplantation (PACT)^29^, Transplant Evaluation Rating Scale (TERS)^30^, and the Stanford Integrated Psychosocial Assessment for Transplantation (SIPAT)^31^. The uses and ramifications of these tools have yet to be widely researched. What is certain is that the lack of a standardized psychosocial assessment and listing criteria allows for inconsistent decision-making, and thus can introduce bias into the patient selection process.

Third, the presence of bias in the delivery of health care has been well established in the literature based on individual characteristics such as gender, age, race, weight, and socioeconomic status^32–37^. Bias can be expressed both explicitly and implicitly. Explicit biases are positive or negative valuations of people based on social characteristics, such as age, gender, sexual orientation, etc. With explicit biases, people are consciously aware of their biases, and they can generally control the expression of their biases to others. Conversely, implicit bias “refers to the attitudes or stereotypes that affect our understanding, actions, and decisions in an unconscious manner. These biases, which encompass both favorable and unfavorable assessments, are activated involuntarily and without an individual’s awareness or intentional control^38^.” All individuals hold implicit biases, and this can directly influence one’s actions without their awareness. There is often a discrepancy between one’s stated values or beliefs, and the implicit biases they hold toward particular groups of people.

For example, one study used the Implicit Association Test (IAT), a tool for measuring implicit bias, to examine physicians’ implicit racial biases and their referral rates for thrombolysis in clinical vignettes of patients with acute coronary syndrome^39^. The study showed that physicians with stronger implicit racial biases were significantly more likely to refer white patients than black patients for thrombolysis, despite comparable clinical data. Though physicians in the study denied explicit racial biases, their behavior demonstrated implicit biases of which they had no awareness. While the majority of the literature on bias in health care focuses on physicians and racial health disparities, any health care professional can hold biases based on a variety of individual characteristics.

Given the extent of bias in health care, it is important to examine the potential for bias in transplantation, particularly with regard to the selection of transplant candidates for the waiting list. There have been few studies on bias in organ transplantation, which have shown racial disparities in access to renal transplantation^40–42^. Such data brings to question whether the lack of national, standardized psychosocial listing criteria allows implicit biases to inadvertently influence decisions made about a patient’s transplant candidacy. Currently, transplant programs create their own psychosocial listing criteria, and they have the option to deviate from those criteria so long as the reasons are documented. This lack of standardization may make listing decisions more vulnerable to providers’ implicit biases, which could lead to unequal access to transplantation.

**Figure 2.**
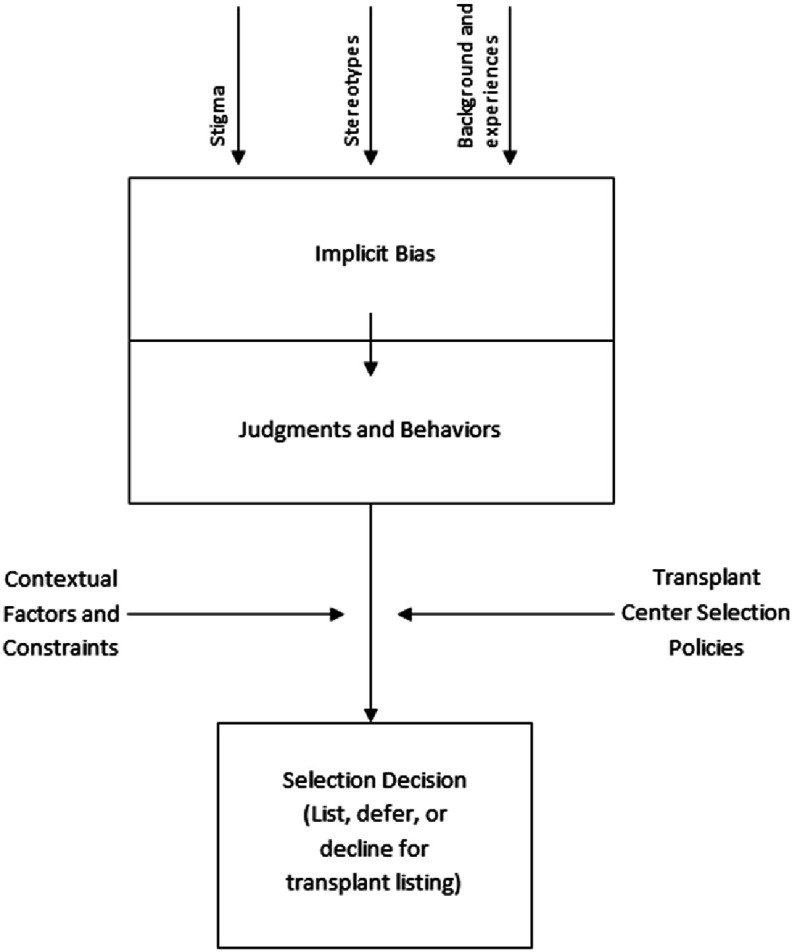
Societal factors, along with the personal background and experiences of a treating clinician, can form implicit biases and potentially influence the selection decision of a transplant candidate.

Figure 2 provides a conceptual depiction of this process, in which societal factors such as stigma and stereotypes, along with the personal background and experiences of a treating clinician, form implicit biases. In turn, implicit biases influence the way in which a clinician views and treats individual patients. Depending on a patient’s social status and background, a clinician’s implicit biases could conceivably privilege or disadvantage a patient in the selection process. A transplant center’s selection policies, and other contextual factors and constraints such as a patient’s medical status, time constraints, and institutional policies and practices, additionally influence selection decisions.

Fourth, we will use two case examples to illustrate the potential for implicit biases to influence the decision-making process for listing patients and the potential for resulting moral distress in transplant staff.

Joe is a 60-year-old with a history of smoking who is undergoing an emergent evaluation for a lung transplant. Joe reports that he has been smoking around five cigarettes per day since the age of 18. Joe reports that he lives in low-income housing and relies on his disability check for income. He shares he has never been married and never had children. He reports that he has a small, but close family. He states that he has “never really liked going to the doctor.” Therefore, he has a significant history of nonadherence to his medical regimen and medical appointments that have resulted in negative health consequences. He states that he will quit smoking and follow his medical regimen, if he receives a lung transplant.

This case illustrates two psychosocial factors that a selection committee would likely consider: (1) nonadherence and (2) nicotine consumption. Below is a review of these factors according to available research.

As previously reviewed in this paper, Joe’s lack of adherence to his medical regimen is a notable risk factor. With regard to smoking, literature has shown that active nicotine consumption is associated with a six-fold increased risk for postoperative pulmonary complications^43^. Moreover, approximately 16% of lung recipients test positive for cotinine post-transplantation^44^. Rates are similar for other transplant populations, with modest estimates ranging from 15% to 25% in some studies^45,46^. In heart transplant patients in particular, smoking six months or less prior to transplantation has been found to be predictive of recidivism for tobacco use^47,48^. Current and past smoking has been further associated with higher rates of morbidity, mortality and allograft loss^49,50,45,46^. However, there is a lack of consensus in the transplant community on current nicotine consumption as a relative versus absolute contraindication to transplantation^10^.

If a transplant selection committee were to review Joe’s case, they would have to determine if his nonadherence and nicotine consumption are prohibitive risks for transplantation. Since transplant centers are provided the flexibility to create their own selection criteria, the decision to list this patient would depend upon the evaluating transplant program’s policies. Thus, considering each risk factor, independently and combined, Joe may or may not be selected for a lung transplant. If the evaluating program has a smoke-free policy for instance, Joe would not be listed unless the program was willing to consider an exception to their policy.

In order to demonstrate the potential bias in the decision-making process, we have changed a few individual characteristics in Joe’s story. To facilitate later comparison of these two cases, we will refer to the patient in this scenario as Andrew.

Andrew is a 45-year-old with a history of smoking undergoing an emergent evaluation for a lung transplant. Andrew reports that he has been smoking around five cigarettes per day since the age of 18. He is a widower raising two young daughters. He lost his job as a medical researcher around the same time that his wife passed away. Andrew knows that smoking is bad for his health, but he continues to smoke as a way to cope with the loss of his wife. He reports that he volunteers for his church and has a network of good friends. He states that he has “never really liked going to the doctor.” Therefore, he has a significant history of nonadherence to his medical regimen and medical appointments that have resulted in negative health consequences. He states that he will quit smoking and follow his medical regimen, if he receives a lung transplant.

In this scenario, Andrew’s medical risks are the same as Joe, but the difference in his social situation is more compelling. Andrew’s smoking and nonadherence behaviors are certainly risk factors for future success in adhering to a strict post-transplant medical regimen. However, Andrew may be viewed in a more favorable light as a transplant candidate than Joe because he has had less years of smoking and presumably has many more years to live. He may also be viewed as a young person who still has the time and opportunity to make changes in his life, which would not be possible if he did not receive a transplant.

This decision to select or deny two similar patients like Joe and Andrew can lead transplant staff to experience moral distress. Moral distress arises when a clinician is unable to take what they view as the right course of action or provide ethically appropriate care due to institutional constraints^51^. In the field of solid organ transplantation, the listing decision can be of great practical and moral consequence. The influence of implicit bias on listing decisions is one aspect that could lead to significant moral distress.

Examining Andrew’s case, members of the transplant team may or may not be aware of the influence that his individual characteristics could have on their recommendations and listing decision. Given Andrew’s compelling social situation, it may be quite stressful for the team to make a decision about his listing status. For instance, if they were to proceed with listing, the team members may be concerned that the patient would not properly care for his donated organ, which is a precious and scarce community resource. They may be concerned about a negative outcome, fearing that the patient may prematurely die as a result of nonadherence behaviors. There may even be concern amongst team members that nonclinical factors, such as the presence of children or his relative youth, should be used to determine his candidacy. Alternatively, if it is determined that Andrew is a not a candidate for a lung transplant, team members may feel like the patient did not receive a fair chance to remediate his risk factors and be alive to raise his children. The lack of clear psychosocial guidelines for transplant listing in this case could result in a lack of consensus in the decision to list the patient and inconsistent decision-making when compared to other similar cases. This could lead to moral distress, negatively affecting team dynamics and morale. Ultimately, this has the potential to inhibit the team’s ability to function optimally and arrive at mutual decisions. The authors have observed this experience at a variety of transplant centers and programs.

As seen in this case example, bias is not restricted to negative perceptions of a patient’s individual characteristics. Bias can also take the form of preferential access to certain medical treatments. Moreover, it should be noted that other factors such as appearance, socioeconomic status, gender, sexuality, alcohol abuse, illicit drug use, profession or social status, ability, etc. could activate implicit biases and further impact selection decisions. In this way, the variability in psychosocial assessments and listing criteria can universally compromise the equitable allocation of donor organs.

## Discussion

This paper has reviewed the non-standardized procedures for the psychosocial selection of lung transplant candidates in the United States. We summarized the current regulatory guidelines from transplant entities and relevant research. Clinical examples were provided to highlight the potential decision-making risks, such as implicit bias and moral distress. This may result from the lack of regulatory standards and best practice guidelines for psychosocial transplant listing criteria.

**Figure 3.**
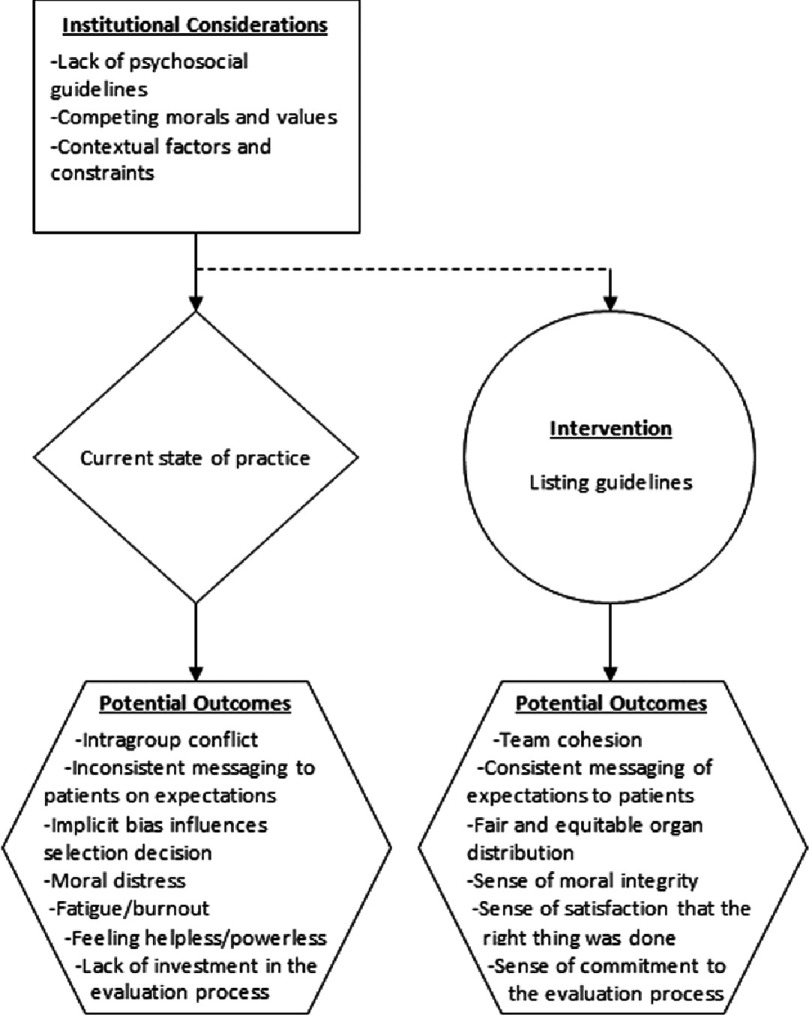
The impact of psychosocial decision-making processes on patient listing. This figure shows both the potential for negative outcomes with the current state of practice, as well as potential positive outcomes stemming from listing guidelines.

In contrast, we propose a semi-structured, standardized approach to the psychosocial assessment and selection of lung transplant candidates. A summary of the institutional considerations discussed in this paper, along with potential outcomes is illustrated in Figure 3. In this figure, standardized psychosocial criteria is offered as one possibility for reducing negative outcomes identified in this paper. Nevertheless, we seek to be clear that the potential positive outcomes described in the figure may not be attained in every patient case and rather are an ideal that programs should continually strive to achieve.

At present, there are assessment tools available to mental health practitioners that may be useful as an initial step towards increased standardization of psychosocial evaluations. These tools include the PACT, TERS, and SIPAT. Our goal is that such tools would support, not replace, clinical decision-making. They are best used to inform treatment plans, interventions, and psychosocial recommendations for listing. Likewise, standardized psychosocial listing criteria could offer parameters for transplant teams to operate within and make informed listing decisions. We envision that this criteria would also include plans for remediation of individual psychosocial risk factors. While patients with different types of organ failure may have a higher prevalence of certain psychosocial risks, the authors believe that psychosocial expectations for transplant patients should be largely uniform. Standardized psychosocial criteria could apply not only to lung patients, but potentially to a variety of solid organ transplant candidates as well. However, the authors acknowledge that there may be some differences in psychosocial considerations based on the type of organ failure.

In conclusion, it is our hope that this introductory review will lead to a larger conversation in the field of transplantation regarding psychosocial practices. We call for standardized psychosocial listing criteria to be applied to the selection of transplant candidates, with the ultimate goal of treating patients consistently and fairly in the listing process. Further research regarding the level of risk that bias poses to patients in the selection process may also be useful in this process. There are current tools, processes, and professional organizations that could help investigate and address these concerns. In the field of transplantation, the ISHLT establishes guidelines for best practices and may be a valuable resource for conducting future research and establishing psychosocial assessment and listing criteria. We recommend further collaboration with other stakeholder groups in order to develop national consensus guidelines about psychosocial transplant listing criteria. Such organizations may include the Society for Transplant Social Workers and the American Society of Transplantation’s Psychosocial Community of Practice.

As a final note, this paper focuses solely on patient-related factors with respect to non-standardization of psychosocial practices. Consequently, the impact non-standardization may have on transplant centers, such as resource utilization, staff allocation, and programmatic finances were not examined. Additionally, the scope of this paper is limited to the United States. The authors therefore recommend an examination of these factors within the purview of future research.

## Competing interests

The authors have no competing interests.

## Funding sources

There are no funding sources for this article.

## Authors’ contributions

Both authors contributed to the conception of the paper, drafting of the manuscript and its revision. All authors read and approved the final manuscript.
